# Deleting IP6K1 stabilizes neuronal sodium–potassium pumps and suppresses excitability

**DOI:** 10.1186/s13041-024-01080-y

**Published:** 2024-02-13

**Authors:** Hongfu Jin, Aili Liu, Alfred C. Chin, Chenglai Fu, Hui Shen, Weiwei Cheng

**Affiliations:** 1 Department of Nuclear Medicine, Xinhua Hospital Affiliated to Shanghai Jiao Tong University School of Medicine, Shanghai, China; 2 Department of Cellular Biology, School of Basic Science, Tianjin Medical University, Tianjin, China; 3 The Solomon H. Snyder Department of Neuroscience, Johns Hopkins University School of Medicine, Baltimore, MD USA; 4 Weill Cornell/Rockefeller/Sloan Kettering Tri-Institutional MD-PhD Program, New York, NY USA; 5 Institute for Developmental and Regenerative Cardiovascular Medicine, Xinhua Hospital, School of Medicine, Shanghai Jiao Tong University, Shanghai, China

**Keywords:** Inositol hexakisphosphate kinase 1, Na^+^/K^+^-ATPase, Neuronal excitability

## Abstract

Inositol pyrophosphates are key signaling molecules that regulate diverse neurobiological processes. We previously reported that the inositol pyrophosphate 5-InsP_7_, generated by inositol hexakisphosphate kinase 1 (IP6K1), governs the degradation of Na^+^/K^+^-ATPase (NKA) via an autoinhibitory domain of PI3K p85α. NKA is required for maintaining electrochemical gradients for proper neuronal firing. Here we characterized the electrophysiology of *IP6K1* knockout (KO) neurons to further expand upon the functions of IP6K1-regulated control of NKA stability. We found that *IP6K1* KO neurons have a lower frequency of action potentials and a specific deepening of the afterhyperpolarization phase. Our results demonstrate that deleting IP6K1 suppresses neuronal excitability, which is consistent with hyperpolarization due to an enrichment of NKA. Given that impaired NKA function contributes to the pathophysiology of various neurological diseases, including hyperexcitability in epilepsy, our findings may have therapeutic implications.

Inositol phosphates constitute a family of key signaling molecules and are phosphorylated to produce higher order members. Inositol hexakisphosphate kinases (IP6Ks) generate 5-diphosphoinositol pentakisphosphate (5-InsP_7_), which contains an energetic pyrophosphate that can bind and/or pyrophosphorylate target proteins. IP6Ks and 5-InsP_7_ regulate a myriad of processes in the brain, including neuronal migration [1, 2], cell death in neurodegenerative diseases [3, 4], synaptic vesicle cycling [5–7], synapse formation in cerebellar Purkinje cells [8], locomotor activity [8–10], social behavior [9], and short-term memory [11]. We previously discovered that IP6K1 and 5-InsP_7_ govern the stability of Na^+^/K^+^-ATPase (NKA) via phosphatidylinositol 3-kinase (PI3K) p85α [12]. Deleting IP6K1 in mice approximately doubles the protein levels of NKA in multiple tissues, including the kidney, heart, and brain. IP6K1 binds PI3K p85α and generates 5-InsP_7_, which binds the RhoGAP domain of PI3K p85α. This disinhibits the interaction between PI3K p85α and NKA, initiating NKA endocytosis and degradation. The increased NKA in the basolateral membrane of renal proximal tubule cells in *IP6K1* knockout (KO) mice was associated with decreased natriuresis in response to a high salt diet.

NKA is critical for establishing electrochemical gradients that enable proper neuronal function. Pumping three Na^+^ out of the cell and two K^+^ into the cell, NKA is required for maintaining the resting membrane potential. NKA pumping accounts for approximately 50% of ATP expenditure in the brain and is the major determinant of neuronal glycolysis activation [13, 14]. NKAs are minimally comprised of a catalytic α-subunit and a regulatory β-subunit with α1/β1 being the most ubiquitously expressed complex [15]. Notably, our previous work found increased α1/β1 NKA in *IP6K1* KO cells [12]. Loss-of-function mutations in *ATP1A1*, which encodes the α1 subunit, cause various diseases including primary aldosteronism, Charcot-Marie Tooth disease, complex spastic paraplegia, hypomagnesemia, and seizures [16].

While pharmacological inhibition of NKA by cardiac glycosides such as digoxin is well-established, there are limited clinical options for therapeutically activating NKA. Boosting NKA levels and/or activity has demonstrated benefits for treating certain neurological disorders such as epilepsy and Parkinson disease. NKA mutations and pharmacological inhibition promote cellular hyperexcitability and drive epilepsy, whereas NKA-activating antibodies were shown to attenuate seizure susceptibility [17]. NKA deficiency aggravates α-synuclein pathology, whereas therapeutic NKA-stabilizing antibodies were shown to ameliorate α-synuclein pathology [18]. Although insulin and β2 adrenergic receptor agonists are used clinically to treat acute hyperkalemia by indirectly increasing NKA expression, they exhibit significant side effects due to their nonspecific mechanisms [19–21]. Recent preclinical studies have demonstrated therapeutic effects of small-molecule IP6K inhibitors [22–24], which may be a targeted strategy to boost neuronal NKA levels for treating neurological disorders such as epilepsy or Parkinson disease.

In this work, we expand the physiological relevance of IP6K1 governing NKA stability by studying the brains of wild-type (WT) and *IP6K1* KO mice. We isolated cerebral cortices and performed western blots (Fig. 1A). NKA levels are approximately 60% higher in cerebral cortices from *IP6K1* KO mice compared to those from WT mice (Fig. 1B,  P = *0.0035*). We further corroborated the increase in cerebral cortex NKA levels via immunostaining (Fig. 1C, D). Our previous work on NKA in kidneys of *IP6K1* KO mice revealed that the increased protein level was accompanied by an increase in total NKA activity [12]. We reasoned that increased NKA levels and pumping activity in the brain would hyperpolarize neurons and suppress neuronal excitability. To characterize functional differences between WT and *IP6K1* KO neurons in the cerebral cortex, we performed electrophysiological studies. The frequency of action potentials in *IP6K1* KO neurons was markedly lower than that of WT neurons (Fig. 1E, F,   P = *0.0039*). Moreover, the hyperpolarization phase of action potentials in *IP6K1* KO neurons was greater in magnitude than that of WT neurons (Fig. 1G).

**Fig. 1:**
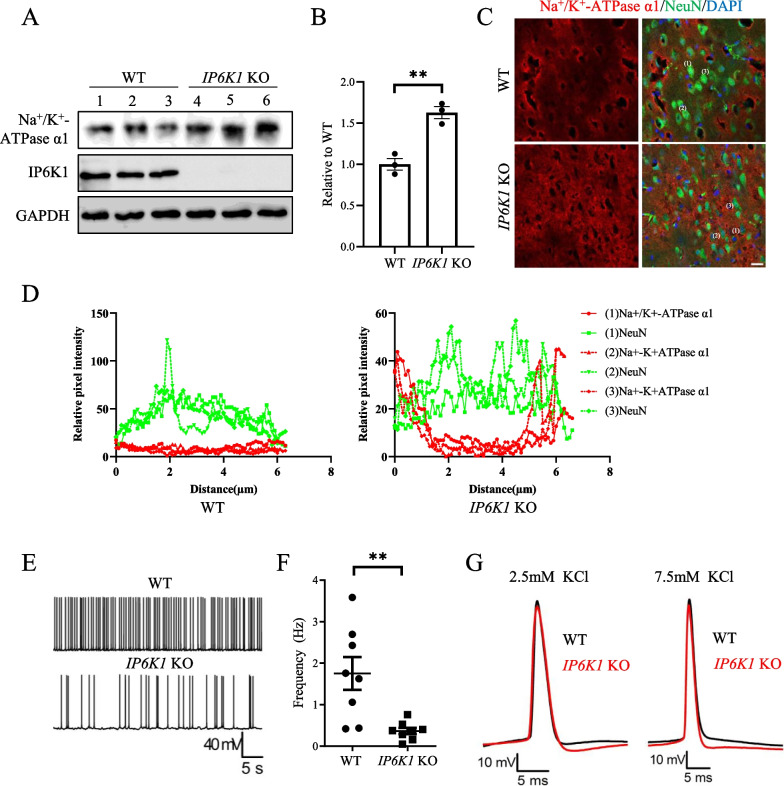
Deleting *IP6K1* increases the expression of Na^+^/K^+^-ATPase-α1, decreases action potential frequency, and expands the hyperpolarization phase in the cerebral cortex of mice. **A**, **B** Western blot and quantification of Na^+^/K^+^-ATPase-α1 protein expression in the cerebral cortex of WT and *IP6K1* KO mice. Na^+^/K^+^-ATPase-α1 expression was increased in *IP6K1* KO mice (n = 3; normalized to GAPDH). **C** Immunostaining showed that Na^+^/K^+^-ATPase-α1 expression of neuronal cells in *IP6K1* KO mice brains was increased. Neuronal nuclei antigen (NeuN) stained mature neuronal cells, and Hoechst 33342 labeled nuclei. Scale bars, 10 μm. **D** Line intensity plots of marked neurons in (**C**) showed that Na + /K + -ATPase-α1 expression was increased in the membrane and cytoplasm of *IP6K1* KO neurons. **E**, **F** *IP6K1* KO neurons displayed diminished action potential frequency. Data represent mean ± SEM, Student’s t-test, n = 8 mice per group. **G** *IP6K1* KO neurons exhibit a higher magnitude of the hyperpolarization phase of the action potential

Our electrophysiological data indicate that deleting IP6K1 reduces neuronal excitability, which is consistent with an enrichment of NKA levels that hyperpolarizes neurons. However, instead of a simple hyperpolarization of the resting membrane potential, we found a specific perturbation of the afterhyperpolarization (AHP) phase of the action potential. AHPs are classified as fast (< 10 ms), medium (~ 50–100 ms), or slow (~ 3–20 s)—fast AHPs follow individual action potentials whereas slow AHPs follow a train of action potentials [25, 26]. AHPs can regulate neuronal firing rates [27]. While AHPs are primarily generated by calcium-gated potassium channels, slow AHPs can be additionally mediated by NKA [26, 28, 29]. Our results show an extended fast AHP and suggest a novel role for NKA beyond regulating slow AHPs, but further studies involving genetic or pharmacological manipulation of NKA in *IP6K1* KO neurons are needed to demonstrate that the perturbations in action potentials are truly mediated by NKA. Because most studies inhibit or delete neuronal NKA, *IP6K1* KO neurons are a unique system to study the physiological effect and therapeutic potential of upregulating NKA in the brain. Lastly, it would be interesting for future studies to investigate whether IP6K1 also regulates cardiac action potentials.

## Methods

### Animals

The IP6K1 WT and KO animals were littermates from heterozygous breeding. All procedures related to animals were performed in accordance with the ethical guidelines of Shanghai Jiao Tong University School of Medicine. Animal experiments were approved by Institutional Animal Care and Use Committee of Xin Hua Hospital Affiliated to Shanghai Jiao Tong University School of Medicine.

### Western blot

The cortex tissues of mice were homogenized and lysed in lysis buffer containing 50 mmol/L Tris–HCl (pH7.4), 100 mmol/L NaCl, 0.5% Igepal CA630, 5 mmol/L MgCl2 and protease/phosphatase inhibitors (Yeasen). Lysates were pulse sonicated and centrifuged at 14,000 *g* for 10 min at 4 ºC. Protein concentrations were normalized using a Pierce BCA Protein Assay Kit (Thermo Fisher Scientific). SDS loading buffer (Thermo Fisher Scientific) containing 5% β-mercaptoethanol was added, and the samples were boiled for 10 min. Proteins were separated by 8–15% SDS-PAGE gel and transferred to a PVDF membrane (Thermo Fisher Scientific). The membrane was blocked with 5% non-fat dry milk in Tris-buffered saline containing 0.1% Tween 20 (TBST) at room temperature (R.T.) for 1 h, and was incubated with primary antibody overnight at 4 °C. The membrane was washed three times with TBST and incubated with HRP-conjugated secondary antibody for 1 h at R.T. followed by three washes with TBST. The blots were detected by the chemiluminescent detection reagents (Thermo Fisher Scientific) along with the ChemiDoc™ imaging system (Bio-Rad). The primary antibodies included Na^+^/K^+^-ATPase-α1 (Proteintech, 14418-1-AP, 1:1000), IP6K1 (Invitrogen, PA5-21531, 1: 1000), GAPDH (Abclonal, AC002, 1:1000).

### Immunofluorescence staining

Animals were perfused and fixed with 4% paraformaldehyde. The brain slices were cut at 25 μm thickness. Slides were washed three times with TBST and blocked with 3% BSA containing 1% goat serum and 0.3% Triton X-100 for 60 min at R.T.. Then the slides were incubated with primary antibodies at 4 °C overnight and washed multiple times with TBST at R.T. Fluorescent-dye conjugated secondary antibodies were added on the slides and incubated for 1 h at R.T.. Nuclei were stained with Hoechst 33342 (Thermo Fisher Scientific) for 5 min. Slices were mounted with ProLong Gold Antifade Mountant (Thermo Fisher Scientific). Images were taken under a confocal microscope (Zeiss LSM 800).

### Electrophysiology

IP6K1 WT and KO mice were anesthetized with isoflurane and quickly decapitated. The brains of mice were rapidly dissected and placed in 0- 4 °C artificial cerebrospinal fluid (ACSF), which contains 2 mM CaCl2, 2 mM MgSO4, 120 mM NaCl, 2.5 mM KCl, 1.25 mM NaH2PO4, 26 mM NaHCO3 and 10 mM Glucose (bubbled with 95% O2 + 5% CO2 gas). Then the brain tissues were fixed on Leica VT1200S Vibratome (Leica, Germany) and cut into brain slices with a thickness of 350 μm. The brain slices were transferred into the ACSF (bubbled with 95% O2 + 5% CO2 gas) at 32 ℃ for one hour and subsequently incubated at room temperature (21–25℃). After incubation, hippocampus CA1 pyramidal neurons were selected under a positive microscope (BX51WI, Olympus, Japan) to perform whole-cell patch-clamp recording.

Recording electrodes (3–5 MΩ resistance) used in this experiment were pulled using P-97 horizontal puller (Sutter, USA). Intra-pipette solution contains 150 mM K-gluconate, 10 mM KCl, 10 mM HEPES, 0.25 mM EGTA, and 5 mM MgATP adjusted to pH 7.2 with KOH. During the entire recording process, the brain slices were completely immersed into ASCF at R.T. and irrigated at a rate of 3 ml/min. The membrane was impaled to form whole-cell configuration after establishing a gigaseal (> 2GΩ). Membrane capacitance and series resistance were compensated by 60–80%. Leakage and capacity currents were also subtracted on-line using a P/4 protocol. Then, the membrane potential was recorded under current clamp mode. The resting membrane potential of *IP6K1* WT and KO neurons were about -64.42 ± 0.36 mV (*P* > *0.05*). Action potential was caused and recorded after KCl was added to the recording bath. Digital signals were obtained with MultiClamp 700B amplifier (Molecular Devices, USA) and Digidata 1440 interface (Molecular Devices, USA). Meanwhile, the recording signals were filtered at 2 kHz and digitized at 10 kHz. All electrophysiology data were acquired and analyzed using pCLAMP10.0 software (Molecular Devices, USA).

### Statistical analysis

GraphPad Prism 9 Software (GraphPad Software Inc., La Jolla, CA) and unpaired two-tailed Student’s t-test were used to perform the statistical analysis. Data are presented as mean ± SEM. The statistical level was 0.05, *P < 0.05, ** *P* < 0.01.

## Data Availability

All data generated and analyzed in the study are included in this article and its supplemental files.

## Abbreviations

IP6K1: Inositol hexakisphosphate kinase 1
NKA: Na^+^/K^+^-ATPase
KO: Knockout
IP6Ks: Inositol hexakisphosphate kinases
5-InsP_7_: 5-Diphosphoinositol pentakisphosphate
PI3K: Phosphatidylinositol 3-kinase
WT: Wild-type
AHP: Afterhyperpolarization
